# Service delivery interventions to increase uptake of voluntary medical male circumcision for HIV prevention: A systematic review

**DOI:** 10.1371/journal.pone.0227755

**Published:** 2020-01-13

**Authors:** Kaitlyn Atkins, Ping Teresa Yeh, Caitlin E. Kennedy, Virginia A. Fonner, Michael D. Sweat, Kevin R. O’Reilly, Rachel Baggaley, George W. Rutherford, Julia Samuelson

**Affiliations:** 1 Social and Behavioral Interventions Program, Department of International Health, Johns Hopkins Bloomberg School of Public Health, Baltimore, Maryland, United States of America; 2 Division of Global and Community Health, Department of Psychiatry and Behavioral Sciences, Medical University of South Carolina, Charleston, South Carolina, United States of America; 3 Department of HIV, World Health Organization, Geneva, Switzerland; 4 Department of Epidemiology and Biostatistics, University of California, San Francisco, San Francisco, California, United States of America; University of the Witwatersrand, SOUTH AFRICA

## Abstract

**Background:**

Voluntary medical male circumcision (VMMC) remains an essential component of combination HIV prevention services, particularly in priority countries in sub-Saharan Africa. As VMMC programs seek to maximize impact and efficiency, and to support World Health Organization guidance, specific uptake-enhancing strategies are critical to identify.

**Methods:**

We systematically reviewed the literature to evaluate the impact of service delivery interventions (e.g., facility layout, service co-location, mobile outreach) on VMMC uptake among adolescent and adult men. For the main effectiveness review, we searched for publications or conference abstracts that measured VMMC uptake or uptake of HIV testing or risk reduction counselling within VMMC services. We synthesized data by coding categories and outcomes. We also reviewed studies assessing acceptability, values/preferences, costs, and feasibility.

**Results:**

Four randomized controlled trials and five observational studies were included in the effectiveness review. Studies took place in South Africa, Tanzania, Uganda, Zambia, and Zimbabwe. They assessed a range of service delivery innovations, including community-, school-, and facility-based interventions. Overall, interventions increased VMMC uptake; some successfully improved uptake among age-specific subpopulations, but urban-rural stratification showed no clear trends. Interventions that increased adult men's uptake included mobile services (compared to static facilities), home-based testing with active referral follow-up, and facility-based HIV testing with enhanced comprehensive sexual education. Six acceptability studies suggested interventions were generally perceived to help men choose to get circumcised. Eleven cost studies suggested interventions create economies-of-scale and efficiencies. Three studies suggested such interventions were feasible, improving facility preparedness, service quality and quantity, and efficiencies.

**Conclusions:**

Innovative changes in male-centered VMMC services can improve adult men’s and adolescent boys' VMMC uptake. Limited evidence on interventions that enhance access and acceptability show promising results, but evidence gaps persist due to inconsistent intervention definition and delivery, due in part to contextual relevance and limited age disaggregation.

## Introduction

As countries strive to control the HIV epidemic, voluntary medical male circumcision (VMMC) remains an essential component of combination HIV prevention, particularly in sub-Saharan Africa. VMMC reduces the risk of HIV acquisition by approximately 60% and also provides an opportunity to link men and boys to a range of other HIV prevention and treatment services [1–3].

VMMC for HIV prevention was introduced in sub-Saharan Africa after the 2007 recommendation by the World Health Organization (WHO) and the Joint United Nations Programme on HIV/AIDS (UNAIDS) to scale-up access to this proven intervention in priority countries where circumcision prevalence was low and HIV prevalence was high [4]. Between 2008 and 2018, over 23 million men and boys were circumcised in priority countries, averting an estimated 230,000 new HIV infections by 2017 and 1.1 million by 2030, accounting for improvements in antiretroviral therapy (ART) coverage [5]. While initial implementation focused on providing VMMC to men in the general population aged 15–49 years, VMMC uptake has been high among adolescent boys [6–9]. As governments and implementing partners respond to lack of coverage among specific subpopulations, programs have used a number of strategies to reach previously unreached populations, including older (adult) men, with VMMC.

Approaches to improve VMMC uptake often include one or a combination of diverse interventions, some of which have been implemented and adopted as good practices in VMMC programs in East and Southern Africa. Despite this, their impact has not been well-documented [10]. The impact of changes to Ministry of Health or VMMC implementing partner service delivery policies and operations (e.g. training, staffing, task-shifting) and physical location (e.g. fixed-site facilities versus mobile clinics) have not been reviewed. To fill this gap, and to provide an evidence base for upcoming WHO guidelines on VMMC, we systematically reviewed the evidence on the effect of service delivery interventions on VMMC uptake among men in East and Southern Africa. A separate review examines economic compensation or incentive-based interventions [11]. We also reviewed the acceptability, values and preferences, costs, and feasibility of service delivery interventions, providing a broader perspective on these types of interventions.

## Methods

We conducted this systematic review in accordance with Preferred Reporting Items for Systematic review and Meta-Analyses (PRISMA) guidelines [12].

### Topic definition

For the purposes of this review, we defined “service delivery interventions” as changes or improvements to the ways VMMC services are provided. Interventions could emphasize additional, restructured, or intensified information, education, or communication services or more structural aspects like improved provider training; changes to physical space; changes to health services structure, such as altering service location or time of day; using home- or community-based services, task-shifting, or restructuring the flow of services; or co-location of VMMC with other health services, such as hypertension or diabetes screening.

### Effectiveness review inclusion criteria

The main question for our effectiveness review was: among adolescent and adult men who are potential candidates for male circumcision for HIV prevention within public health VMMC programs, do service delivery interventions lead to improved uptake of male circumcision compared to no such interventions, or a different or less intensive type of intervention? We followed the PICO question format to define our review criteria:

- **Population:** Uncircumcised adolescent and adult men (ages 10+) who are potential candidates for male circumcision for HIV prevention within public health VMMC programs
- **Intervention:** Service delivery interventions
- **Comparison:** No such service delivery interventions, or a different or less intensive type of service delivery intervention
- **Outcomes:** Primary: 1) Uptake of VMMC for HIV prevention as defined by WHO; Secondary: 2) Uptake of HIV testing within VMMC services, and 3) Uptake of safer sex and risk reduction counselling within VMMC services

In addition, to be included in the review, a study must have: 1) had a study design that presented a pre/post or multi-arm comparison by service delivery intervention exposure, 2) measured one or more of the outcomes listed above, and 3) been published in a peer-reviewed journal or conference abstract. No restrictions were placed based on type or scope of service delivery intervention, location of the intervention, or language of the publication. When information was available for the same study from both a peer-reviewed article and a conference abstract, we only used data from the peer-reviewed article.

When possible, outcomes were stratified by whether participants (men) were considered high-risk for HIV or not, since recent modeling suggests VMMC may have greater impacts among men at increased risk for HIV [13]. We defined high-risk as having any one of the three following characteristics: 1) being in the three highest HIV incidence 5-year age strata per UNAIDS country estimates, 2) having >1 sexual partner (lifetime, current, or within a specified timeframe) [14,15], and/or 3) having a recent history of a sexually transmitted infection (STI) other than HIV. Outcomes were also stratified by the following age groupings: younger adolescents aged 10–14 years, older adolescents aged 15–19 years, and adults aged ≥20 years. If data were available, we further age-stratified the adult population as well. These age categories are those used by the Decision Makers Program Planning Toolkit [7,16], which suggests tailored VMMC platforms by age; we felt that different service delivery strategies might be relevant for different age groups (e.g., school-based programs for adolescents, or extended clinic hours for working-aged men) or that we might need to consider specific risks and benefits of VMMC by age group. Finally, we employed a health equity lens by stratifying where possible by PROGRESS variables: place of residence (e.g. rural/urban); race, ethnicity, culture, and language; occupation; gender/sex; religion; education; socioeconomic status; and social capital [17].

### Search strategy and screening

The search was developed in conjunction with another related review on economic compensation interventions to increase uptake of VMMC developed for the same WHO guidelines [11]. We therefore kept the search strategy broad, so as to include articles relevant to both topics.

We searched five electronic databases (PubMed, CINAHL, Sociological Abstracts, PsycINFO, and EMBASE) and four conferences (International AIDS Conference (IAC), International AIDS Society (IAS) Conference on HIV Science, Conference on Retroviruses and Opportunistic Infections (CROI), and International Conference on AIDS and STIs in Africa (ICASA)). Electronic databases were searched from January 1, 1990 through May 31, 2018. The IAC, IAS, and ICASA conference abstracts were searched for all available years (through mid-2018). For CROI, only the most recent conferences (2014–2018) were searched as past conference abstracts are inaccessible. We searched the bibliographies of all studies included in the review and of a previous related review [18]. Further, selected experts in the field–specifically, members of the WHO Guideline Development Group for Medical Male Circumcision–were asked to share additional articles not identified through other search methods. We also searched for ongoing RCTs through clinicaltrials.gov, the WHO International Clinical Trials Registry Platform, the Pan African Clinical Trial Registry, and the Australian New Zealand Clinical Trials Registry.

The following search strategy was adapted for entry into all electronic databases: (HIV [tiab] OR “human immunodeficiency virus” [tiab]) AND (circumcision [tiab] or circumcis* [tiab] or VMMC [tiab]). Only the term “circumcision” was used to search conference abstracts because all conferences searched are HIV-related and search functions are limited.

Titles, abstracts, citation information, and descriptor terms of citations identified through the search were screened in duplicate. Full-text articles were obtained of all selected abstracts, and two independent reviewers assessed all full-text articles for eligibility to determine final study selection. Differences were resolved through consensus.

### Data extraction and management

Data were extracted independently in duplicate using standardized data extraction forms. Differences in data extraction were resolved through consensus and referral to a senior study team member when necessary. The following information was gathered from each included study:

- Study identification: Author(s), type of citation, year of publication
- Study description: Study objectives, location; population characteristics, main intervention description (for all study arms), description of any additional intervention components, study design, sample size, follow-up periods, loss to follow-up
- Outcomes: Analytic approach, outcome measures, comparison groups, effect measures and sizes, confidence intervals, significance levels, conclusions, limitations

For randomized controlled trials (RCTs), we assessed risk of bias using the Cochrane Collaboration’s tool [19]. We assessed methodological components of the studies and classified studies as having high or low risk of bias. For studies that were non-randomized but comparative, we assessed study rigor using the adapted Evidence Project Risk of Bias tool for intervention evaluations [20].

### Data analysis

We analyzed data according to coding categories and outcomes. Given the diverse intervention approaches and different ways of reporting outcomes, we were unable to combine effect sizes in meta-analysis. The terms 'effectiveness' and 'impact' are used throughout to refer to findings from the entire included body of literature, including observational studies which do not prove causality, to match the programmatic use of the terms in the WHO data collection process for which this review was performed.

### Additional reviews

In addition to the effectiveness review, we conducted additional reviews assessing acceptability, values and preferences, costs, and feasibility of service delivery interventions. These reviews added important, complementary evidence for the purpose of developing holistic WHO guidelines [21]. We used the same search strategy to identify studies for these reviews. These studies could be qualitative or quantitative in nature but must have presented primary data collection; opinion pieces, editorials, and review articles were not included. We summarized this literature qualitatively and organized it by study design, methodology, location, and population.

#### Acceptability review

We included studies in the acceptability review if they presented primary data examining people’s feelings about the acceptability of the interventions included in the main effectiveness review. We focused on studies examining the perspectives of men who have used or potentially would use VMMC, but we also included studies examining the acceptability of the interventions among providers and other stakeholders (such as partners, families, and communities).

#### Values and preferences review

We included studies in the values and preferences review if they presented primary data examining people’s feelings about the three PICO outcomes: 1) uptake of medical male circumcision, 2) uptake of HIV testing within VMMC services, and 3) uptake of safer sex and risk reduction counselling within VMMC services. We assessed values and preferences around population-level uptake of circumcision as an outcome, rather than feelings about circumcision itself as a procedure. We were primarily interested in studies examining the perspectives of people who have used or potentially would use VMMC, but we also included studies examining values and preferences among providers and other stakeholders (such as partners, families, communities, and policy-makers).

#### Costs review

We included studies in the costs review if they presented primary data examining cost or cost-effectiveness of the service delivery interventions, or otherwise discussed resource use in relation to these interventions. We considered any measure of cost (total cost, unit cost, etc.).

#### Feasibility review

We included studies in the feasibility review if they presented primary data examining issues with provision of the service delivery interventions or how they fit within health systems (training, monitoring, evaluation, etc.). This was intended to complement the effectiveness review by identifying issues related to implementation of these interventions that might be relevant for others seeking to replicate them.

### Data availability

All data came from published journal articles or conference abstracts.

## Results

### Effectiveness review

**Fig 1:**
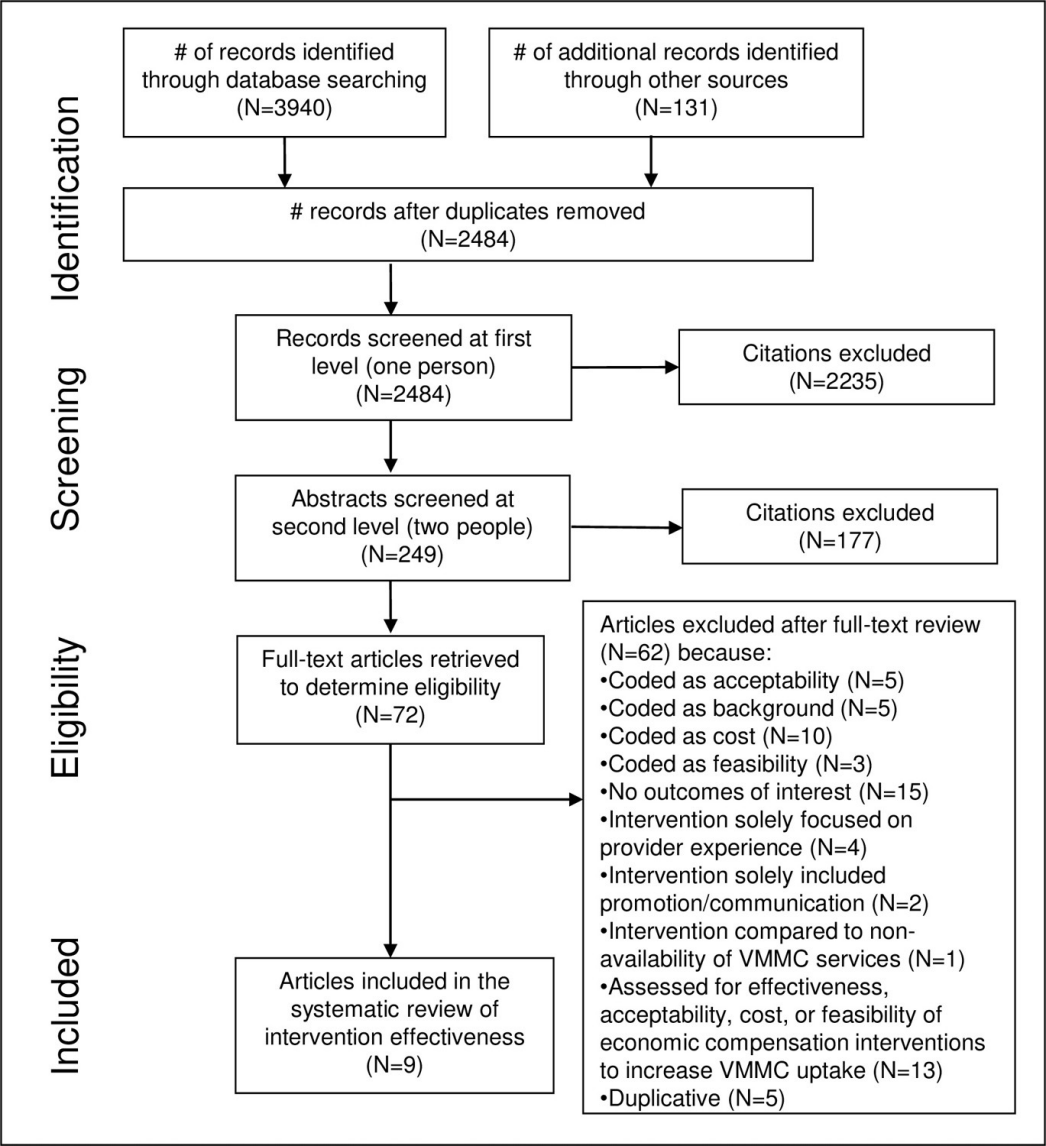
Flow of information through the different phases of a systematic review.

Electronic database searching retrieved 3,940 results, and an additional 131 were identified through searching conference databases, trial registries, contacting experts in the field, and secondary searching (Fig 1). After removing duplicates, there were 2,484 unique citations. After initial screening of titles and abstracts, 249 citations remained. After two independent reviewers screened in duplicate and gained consensus, we selected 72 articles for full-text review. Of these, we included nine studies in the effectiveness review [22–30].

Of the nine included studies in the effectiveness review, four were RCTs [23,25,29,30], and five were observational studies, either cross-sectional studies with multiple groups for comparison or serial cross-sectional studies comparing before and after service delivery intervention implementation [22,24,26–28]. Studies took place in South Africa [23,28], Tanzania [22,24,26,29], Uganda [23,27], Zambia [30], and Zimbabwe [22,25]. Studies included adult men and adolescent boys aged 10 years and older [22,29], 13 years and older [27], 18 years and older [23,24,26,30], 14–20 years [25], and 10–49 years [28], though some specifically targeted adolescent male students [25,27,28] or adult men (15–49 years [22], 18 years or older [30], or 20–34 years [29]). Intervention approaches varied widely and were often multi-component, so we grouped them from a programme implementer perspective based on delivery platforms: community- (including home-)based interventions [22–24,26,29], school-based interventions [25,27,28], and facility-based interventions [30]. Table 1 presents a summary description of included studies; results for each study are presented in Table 2.

**Table 1:** Description of included studies–effectiveness review.

Study	Location, Population, Study design	Service delivery intervention	Comparison
**Community-based**			
Ashengo et al., 2014 [22]	Tanzania, Zimbabwe (rural) Adolescent boys & men aged 10+ (n = 173,809) Cross-sectional	VMMC campaign: high volume VMMC services with task-shifting and increased promotional activities, for a short period of time, both at fixed sites (co-located within existing health care facilities at central, provincial, or district levels or at stand-alone sites) and outreach sites (mobile VMMC units visit lower level facilities and clinics, clinics at workplaces in mining/farming areas, schools, etc.)	Routine facility-based VMMC
Barnabas et al., 2016 [23]	South Africa, Uganda (rural) Men (n = 750) RCT	Following home-based HIV testing services (HTS), different VMMC referral/reminder approaches: **Intervention 1:** VMMC text message reminders (i.e. receive "this could be the best decision you make–act now!" text message three weeks after HTS and follow-up phone call 1 month after HTS; if men had not gotten circumcised by the one-month follow-up call, then text message 6–7 weeks after HTS and phone call two months after HTS) **Intervention 2:** Lay counsellor follow-up home visits to promote VMMC	Following home-based HTS, received standard clinic referral card for VMMC
Hellar et al., 2015 [24]	Tanzania (rural) Men (n = 148,880) Cross-sectional	**Intervention 1:** VMMC outreach campaign (teams of providers move into new communities and do 1–3 week bursts of intense high-volume mobile/facility VMMC services with increased promotional activities) **Intervention 2:** mobile VMMC services (target rural, hard-to-reach areas with a roving team of providers, often providing services in non-facility settings and at lower volumes than campaigns)	Routine facility-based VMMC
Mahler et al., 2015 [26]	Tanzania (rural) Men (n = 267,917) Serial cross-sectional	GIS (geographic information systems technology, i.e. interactive digital maps) as a decision-making tool to strategically plan and implement VMMC mobile outreach / campaigns to reach lesser-served areas (i.e. rural areas) through providing services at local dispensaries and non-health-care facilities	No intervention
Wambura et al., 2017 [29]	Tanzania (rural) Adolescent boys & men aged 10+ (n = 10,117) RCT	Intensified demand-creation intervention package (enhanced public address messages, peer promotion by recently circumcised men, PLUS outreach ‘parent’ and satellite sites along with service facility changes to increase privacy, and engagement and education of female partners) in the context of VMMC outreach services	Standard VMMC outreach services (without additional demand-creation intervention)
**School-based**			
Kaufman et al., 2016 [25]	Zimbabwe (urban) Adolescent male secondary school students age 14–20 (n = 1,226) RCT	"Make the Cut (MTC)(+)" delivered through schools: Trained “coach” (circumcised man aged 18–30 years) facilitated interactive game (metaphor for HIV protection), shared a personal story, and led group discussion about VMMC; coach followed up with students and facilitated transport to VMMC clinic	No intervention
Miiro et al., 2017 [27]	Uganda (urban) Male students age 13+ (n = 127) Serial cross-sectional	Modification of MTC soccer-based promotion in schools to include home visits, a video to facilitate discussion of VMMC with boys’ parents, closer coordination with schools, and rescheduling of activities to accommodate exams and holidays. .	MTC without modifications: Trained “coach” (circumcised man aged 18–30 years) facilitated interactive game (metaphor for HIV protection), shared a personal story, led group discussion about VMMC, accompanied students to VMMC clinic, and provided follow-up.
Montague et al., 2014 [28]	South Africa (rural) Male high school students, but also open to younger/ older community members, age 10–49 (n = 11,088) Serial cross-sectional	Phased approach of school-based VMMC promotion and service access. Community sensitization meetings. In-school VMMC awareness sessions, teacher liaison, and VMMC coordinators. VMMC and HTS services offered in schools. Schedule optimization: First HTS on Monday through Thursday during school hours and VMMC on Friday and Saturday to minimize school disruption, then both services on Fri-Sat. Peer recruitment to VMMC services, including scheduling appointments and coordinating transport (with incentives for peer recruiters). Post-operative services provided in-school. HIV positive linked to care.	Community consultation/ engagement and routine facility-based VMMC
**Facility-based**			
Weiss et al., 2015 [30]	Zambia (urban) Men (n = 800) RCT	Spear and Shield: recruited after HTS at community health centers. Four session group-based comprehensive educational program about HIV/sexual risk reduction and male circumcision promotion, including condom negotiation, cognitive-behavioral skills-building, peer mentoring/coach who has undergone VMMC, and VMMC education. Engagement/invitation to female partners. Training of health care providers to implement above educational sessions.	Video-based attention-control group educational sessions (time-matched) on endemic disease prevention strategies (tuberculosis, malaria, cholera, and water-borne diseases) and condom provision

HTS, HIV testing services; MTC, Make the Cut.

**Table 2:** Comparative results from included studies–effectiveness review.

Study	Uptake of VMMC: Overall	Uptake of VMMC: Stratified analyses	Uptake of HIV testing in VMMC services
**Community-based**			
Ashengo et al., 2014 [22]	**Number of VMMC procedures performed (over 38 months)** Comparison: 68,777 (18,261 Zimbabwe; 50,516 Tanzania) Intervention: 105,032 (45,435 Zimbabwe; 59,597 Tanzania) In Zimbabwe, significantly more male circumcisions were done during campaigns than during routine service delivery, 64% vs. 36% respectively (p<0.00001), but no statistically significant difference in Tanzania (p>0.05).	In Tanzania: For all age groups, with the exception of those above the age of 35, clients attending campaign services were significantly younger than those attending routine services (p = 0.0001) in both campaign and routine service delivery combined. Majority of clients served during campaign (59%) were in the 10–14 year age group; The majority of clients served during routine service delivery (64%) were above 15 years of age (p<0.0001). In Zimbabwe: The proportions of males from the age group 10–14, 15–19, and 20–24 years accessing VMMC services during campaigns were significantly higher than those accessing routine services (p < .05). However, for men above 25 years, the difference between campaign and routine services are not significantly different. Those aged 20–24 (48% campaign vs. 52% routine), 25–29 (45% campaign vs. 55% routine), 30–34 (45% campaign vs. 55% routine), and 50 years and above (47% campaign vs. 53% routine) were reached almost evenly through campaign (mainly outreach) and routine (mainly fixed) service delivery.	
Barnabas et al., 2016 [23]	**VMMC uptake (at 3-month follow-up)** Control: 62/224 (28%) Intervention 1 (text message): 137/284 (48%), RR: 1.72 (95% CI: 1.36 to 2.17), p<0.0001 Intervention 2 (lay counsellor follow-up): 106/226 (47%), RR: 1.67 (95% CI: 1.29 to 2.14), p = 0.0001		
Hellar et al., 2015 [24]	**Number of VMMC procedures performed (over 9 months)** Routine: 11,392 Campaign: 132,080 Mobile: 5,408	Mobile teams reached more older clients compared to other service modalities (p<0.001) Routine: 11,392 clients, 71% clients <20 years, 29% clients ≥20 years Campaign: 132,080 clients, 78% clients <20 years, 22% clients ≥20 years Mobile: 5,408 clients, 62% clients <20 years, 38% clients ≥20 years Total: 148,880 clients, 76% clients <20 years, 24% clients ≥20 years	**Onsite HIV testing rate** Routine: 10,992/11,392 (96.5%) Campaign: 128,801/132,080 (97.5%) Mobile: 5,129/5,408 (94.8%) Overall uptake high (97.3%) regardless of modality. X^2^ = 246.0565, p <0.001
Mahler et al., 2015 [26]	**Number of VMMC procedures (per 12-month period)** Baseline: FY2010: 22,970; FY2011: 42,667 Follow-up (using GIS to prioritize VMMC scale-up): FY2012: 49,949; FY2013: 64,407; FY2014: 87,924		
Wambura et al., 2017 [29]	**Mean number of VMMC clients per cluster (over 1 month)** Control: 393 (SE: 83) Intervention: 619 (SE: 110) Mean difference: 227 (95% CI: 33 to 420), p = 0.03	**Proportion of clients aged 20–34 years** (I: 17.7% vs C: 13.0%, PR = 1.36, 95% CI: 0.93–1.98, p = 0.11) *Mean number of clients aged 20–34 years (I: 146, SE: 42 vs C: 49, SE: 10, Mean difference: 97, 95% CI: 40–154, p = 0.002) *Proportion of clients aged ≥20 years (I: 25.5% vs C: 16.0%, PR = 1.60, 95% CI: 1.09–2.34, p = 0.02) *Mean number of clients aged ≥20 years (I: 193, SE: 50 vs C: 60, SE: 12, Mean difference: 133, 95% CI: 59–207, p = 0.002) **Proportion of clients aged 20–29 years** (I: 14.5% vs C: 11.0%, PR = 1.31, 95% CI: 0.88–1.95, p = 0.16) *statistically significant	**VMMC clients accepting HIV testing** Control: 3,902/3,926 (99.4%) Intervention: 6,072/6,191 (98.1%) RR: 0.987 (0.983, 0.991)
**School-based**			
Kaufman et al., 2016 [25]	**VMMC uptake (at 4-month follow-up)** Control: 17/371 (4.6%) Intervention: 37/304 (12.2%) Difference in proportions: 7.6% OR: 2.65 (95% CI: 1.19 to 5.86), p = 0.01	Intervention associated with greater VMMC uptake in younger age groups: • 14–15 years: I: 13/126 (10.3%) vs C: 5/144 (3.5%), 6.8% difference, OR 2.99 (95% CI: 0.94–9.53), p = 0.06 • 16–17 years: I: 20/149 (13.4%) vs C: 7/189 (3.7%), 9.7% difference, OR 4.19 (95% CI 1.70–10.3), p = 0.002 • ≥18 years: I: 4/26 (15.4%) vs C: 5/25 (20.0%), -4.6% difference, OR 0.88 (95% CI: 0.16–4.65), p = 0.88	
Miiro et al., 2017 [27]	**VMMC uptake (timeframe not specified)** Comparison: 6/58 (10%) Intervention: 16/69 (23%)		
Montague et al., 2014 [28]	**Number of VMMC procedures (per month)** Baseline: 58 VMMCs/month 21-month follow-up: 308 VMMCs/month		
**Facility-based**			
Weiss et al., 2015 [30]	**VMMC uptake (at 12-month follow-up)** Control: 96/396 (24.2%) Intervention: 161/389 (41.4%) OR: 2.45 (95% CI: 1.24 to 4.90), p = 0.02		

C, comparator; CI, confidence interval; GIS, geographic information systems; I, intervention; OR, odds ratio; PR, prevalence ratio; RR, relative risk; SE, standard error; VMMC, voluntary male medical circumcision.

Quality assessment of included studies are summarized in S1 Table. The four RCTs included in this review assessed two community-based interventions, one facility-based intervention, and one school-based intervention [23,25,29,30]. These studies had low risk of bias. Although the four RCTs were not blinded, we judged them to have low risk of performance or detection bias, as lack of blinding was unlikely to influence uptake outcomes. One RCT was found to have high risk of other bias, due to technical issues during implementation that resulted in few participants receiving in the intervention after the first randomization and subsequent re-randomization for adequate power [23]. Five non-randomized studies assessed the impact of three community-based and two school-based interventions and showed low to medium risk of bias [22,24,26–28]. Two studies did not report pre-/post-intervention data [22,24], and one did not include comparison groups [26]. Of those studies with comparison groups, none assessed differences between study groups in measured baseline variables [22,24,27,28].

#### Uptake of VMMC

Results regarding VMMC uptake from community-, facility-, and school-based service delivery interventions are summarized in Table 3. All included studies reported that service delivery interventions were associated with improved VMMC uptake; however, the magnitude of effect varied.

**Table 3:** Summary of effects.

	Number of patients with the desired outcome/total sample size (%)		Effect	
No. of studies	Service delivery interventions	Comparison group	Relative (RR) (95% CI)	Absolute (RD) (95% CI)
**Uptake of VMMC**				
***Community-based—RCTs (follow up: 3 months)***				
2 ^a^^,^^b^ [23,29]	106/226 (46.9%)	62/224 (27.7%)	**1.67** (1.29 to 2.14)	**185 more per 1,000** (80 more to 316 more)
***Community-based—Observational***				
3 [22,24,26]	Studies were not pooled, but generally reported more circumcisions associated with service delivery interventions.			
***Facility-based—RCTs (follow up*: *12 months)***				
1 [30]	161/389 (41.4%)	96/396 (24.2%)	**1.71** (1.38 to 2.11)	**172 more per 1,000** (92 more to 269 more)
***School-based—RCTs (follow up*: *4 months)***				
1 [25]	37/304 (12.2%)	17/371 (4.6%)	**2.66** (1.53 to 4.62)	**76 more per 1,000** (24 more to 166 more)
***School-based—Observational***				
2 ^c^ [27,28]	16/69 (23.2%)	6/58 (10.3%)	**2.24** (0.94 to 5.36)	**128 more per 1,000** (6 fewer to 451 more)
**Uptake of HIV testing and counseling within VMMC services**				
***Community-based—RCTs (follow up*: *3 months)***				
1 ^d^ [29]	6072/6191 (98.1%)	3902/3926 (99.4%)	**0.99** (0.98 to 0.99)	**10 fewer per 1,000** (from 10 fewer to 20 fewer)
***Community-based—Observational***				
1 [24]	128801/132080 (97.5%)	10992/11392 (96.5%)	**1.01** (1.01 to 1.01)	**10 more per 1,000** (from 10 more to 10 more)

CI: confidence interval; RR: relative risk; RD: risk difference; VMMC: voluntary male medical circumcision.

a. Data from Barnabas et al., 2016, only. Additional data from Wambura et al., 2017, on mean number of VMMC clients per cluster (not presented in GRADE table due to lack of denominator for the outcome of interest): Intervention: 619 (standard error: 110) Control: 393 (standard error: 83), Mean difference: 227 (95% CI: 33 to 420), p = 0.03.

b. Data from Barnabas et al., 2016 presented for most intensive (lay counselor follow-up) vs. least intensive (standard referral) comparison.

c. Additional data from Montague et al., 2014 was not presented in GRADE table due to lack of denominator for the outcome of interest: VMMC procedures per month: Pre-intervention: 58 VMMCs/month vs Post-intervention: 308 VMMCs/month.

d. Original publication did not provided data separated by study group; additional data provided by study authors.

Evidence from two community- (including home)-based RCTs showed increased VMMC among participants in service delivery interventions [23,29]. One RCT assessing active referral follow-ups after initial home-based visits for HIV testing in rural South Africa and Uganda found that, compared to standard (written) referral, providing text message and phone call reminders to HIV-uninfected men after HIV testing increased VMMC uptake (relative risk (RR): 1.67, 95% confidence interval (CI): 1.29 to 2.14; risk difference (RD): 185 per 1,000, 95% CI: 80 to 316) [23]. Another RCT in Tanzania found increased uptake in community sites randomized to receive enhanced demand creation, female partner engagement, and increased facility privacy including through expanded outreach compared to sites receiving standard VMMC outreach, when comparing the mean number of VMMC clients per cluster per month between intervention and control (mean difference: 227, 95% CI: 33 to 420, p = 0.03) [29]. Data from the three community-based observational studies, which assessed making services more accessible through periodic VMMC campaigns, mobile services, and targeting of services to underserved areas by using geographic information systems (GIS), were not pooled, but similarly reported increased VMMC following implementation of service delivery interventions, compared to routine pre-intervention facility-based services [22,24,26]. In Zimbabwe, VMMC uptake was significantly higher during periodic outreach campaigns (64%) than during routine services (36%) (p<0.001) over a 38-month period [22]. Campaigns in Tanzania over 9 months resulted in 132,080 VMMC procedures, compared to routine facilities (11,392) or mobile services (5,408) [24]. Using GIS to target VMMC service delivery in Tanzania reached more clients in rural areas, with 48% of VMMCs performed in rural areas before GIS targeting, 88% in rural areas in the year immediately following program implementation, and 93% by three years post-intervention [26].

One facility-based RCT examining VMMC uptake at 12-month follow-up found increased likelihood of VMMC uptake among men receiving the Spear and Shield intervention, a four-session HIV/sexual risk reduction education and VMMC promotion program with specialized training for healthcare providers (RR: 1.71, 95% CI: 1.38 to 2.11; RD: 172 per 1,000, 95% CI: 92 to 269) compared to those who participated in time-matched educational sessions on tuberculosis, malaria, cholera, and water-borne disease prevention and received condoms [30].

Finally, one RCT and two observational studies reported data on VMMC uptake following school-based interventions [25,27,28] targeting both men and boys but focusing on adolescents. The RCT in Zimbabwe reported that male students involved in the "Make The Cut+" program (using a trained coach to spark discussion on VMMC, follow-up with students, and facilitate transport to the VMMC clinic) were 2.66 times more likely to be circumcised (95% CI: 1.53 to 4.62), with an absolute difference of 76 per 1,000 (95% CI: 24 to 166) [25]. Two serial cross-sectional studies showed that men and boys receiving school-based interventions (i.e. soccer-based promotion by VMMC champions who accompanied participants to VMMC and provided follow-up; schedule optimization, peer recruitment, and transportation coordination; parent and community engagement) increased VMMC uptake: in Uganda, they were 2.24 times more likely to be circumcised (95% CI: 0.94 to 5.36), an absolute difference of 128 more per 1,000 (95% CI: 6 fewer to 451 more), and in South Africa, the number of VMMC procedures per month increased from 58 pre-intervention to 308 post-intervention [27,28].

#### Uptake of VMMC: Stratified analyses by age and setting

Multiple studies included adolescents and young men but did not stratify outcome data by age. Several school-based studies included older men, but participants were predominantly less than 20 years old. Exposure to a community-based demand-generation intervention was associated with greater mean number of clients aged 20–34 and a greater proportion of clients aged ≥20 over a one-month period [29]. The proportions of males from the age groups 10–14, 15–19, and 20–24 years accessing VMMC services during campaigns were significantly higher than those accessing routine services (p<0.05) [22]. In Tanzania, mobile VMMC services were found to reach older clients compared with campaigns or routine service delivery (p<0.001); 38% of mobile VMMC clients were aged 20 years or older, compared with 22% and 29% of clients in campaigns and routine services, respectively [24]. A demand-generation program with facilitated linkage to VMMC services for secondary school students was associated with greater VMMC uptake in younger age groups (14–17 years old) compared with adults (≥18 years) [25].

Three included studies took place in urban areas and six in rural settings; however, there were no clear trends in VMMC uptake by location. One study presented additional data comparing uptake before and after rollout of GIS prioritization of VMMC services; after data-driven programmatic prioritization, VMMC procedures shifted from those primarily conducted in urban settings (around 60%) before GIS to almost all in rural settings (around 90%) afterward [26].

#### Uptake of HIV testing and counseling within VMMC services

Two studies in Tanzania (one RCT and one comparative cross-sectional study) measured uptake of HIV testing in VMMC services, comparing service delivery approaches [24,29]. Authors of the RCT provided additional data stratified by study arm which was not available in the original article. In both studies, participants demonstrated high acceptance of HIV testing within VMMC services, ranging from 96.5% to 99.4% uptake across arms. No meaningful difference in uptake of HIV testing was observed (Table 3). Neither study stratified uptake of HIV testing.

#### Uptake of safer sex and risk reduction counseling within VMMC services

No data were found comparing exposure versus non-exposure to service delivery interventions on uptake of safer sex and risk reduction counseling within VMMC services.

### Acceptability

We identified six studies examining acceptability of service delivery interventions (Table 4) [27,31–35], one of which was also included in our main effectiveness review [27] and another which was related to a study presented in the effectiveness review [31]. Studies were published between 2012 and 2018 and took place in Botswana [32,35], South Africa [34], Tanzania [33], Uganda [27], and Zimbabwe [31]. These studies captured the perspectives of (potential) users of VMMC and other stakeholders (e.g. providers, partners, families, communities) on aspects of service delivery interventions that increased the acceptability and uptake of VMMC. Three of the included studies assessed the acceptability of community-based interventions [32–34], two assessed school-based interventions [27,31], and one assessed a facility-based intervention [35].

**Table 4:** Description of included studies–acceptability review.

Study	Location	Intervention	Methods and participants	Acceptability findings
**Community-based**				
Katisi et al., 2015 [32]	Botswana	VMMC demand creation campaigns and community consultation procedures	Participant observation, qualitative interviews, and focus groups Interviews and focus groups with leaders in partner organizations, implementers, social workers, traditional leaders, and men in communities. Observations of partner organization meetings, VMMC marketing campaigns.	There were conflicting views on public VMMC demand creation campaigns in relation to the traditional secrecy of male circumcision and lack of consultation with community leaders. HIV testing in VMMC was considered a barrier to getting men to circumcise.
Kilima et al., 2012 [33]	Tanzania	Involving traditional practitioners in VMMC scale-up	Quantitative survey and qualitative interviews 601 households surveyed. Interviews with national and district health officers, health care providers, and traditional healers.	Nearly all participants were in favor of the move to promote a partnership between conventional and traditional circumcision practitioners, and involving traditional practitioners in scaling up male circumcision in the context of HIV prevention.
Marshall et al., 2017 [34]	South Africa	Household: flyer and discussion about VMMC Individual: motivational interview and follow-up with male circumcision adviser	Quantitative survey 142 men undergoing VMMC	81.7% said discussions with the male circumcision adviser were very important, and 83% reported that they would not have been circumcised without these discussions. 85.2% said the flyer was very important.
**School-based**				
DeCelles et al., 2016 [31] (related to Kaufman et al., 2016 [25] in effectiveness review)	Zimbabwe	“Make the Cut”: Trained “coach” facilitated interactive game, shared a personal story, and led group discussion about VMMC; coach followed up with students and facilitated transport to VMMC clinic	Qualitative interviews and focus groups 17 interviews and 2 focus groups with coaches and 29 interviews with circumcised (n = 13) and uncircumcised boys (n = 16)	Findings suggested acceptability of "Make the Cut" curriculum components. Participants cited the Coach’s Story as a motivational component. They valued their coaches, particularly their openness and honesty when discussing VMMC. They trusted their coaches and relied on them for support. Coaches shared similar feelings about the importance of the relationships with participants, particularly if they were recommending VMMC. Participant age posed challenges, as coaches felt older participants were less interested in both soccer and VMMC.
Miiro et al., 2017 [27] (also in effectiveness review)	Uganda	“Make the Cut” with added home visits, a video, closer coordination with schools, and accommodation of exams and holidays	Qualitative interviews 5 circumcised boys and 5 uncircumcised boys	Circumcised boys had social support for VMMC from family and peers in the form of information and encouragement. They said the decision to circumcise was prompted by coaches, who played a crucial role in the explanation of information regarding circumcision including the healing process and the discussion of the myths or misconceptions that had prevented boys from deciding to circumcise.
**Facility-based**				
Semo et al., 2018 [35]	Botswana	Health system changes to improve VMMC uptake	Focus groups 27 focus groups with men (stratified by circumcision status and age), women (stratified by age), and community leaders	Participant suggestions included maximize access to VMMC services through reduction of logistic hurdles (distance to facilities, wait times, and fees), bring VMMC services closer to people through mobile trucks or offering at all health facilities (not just a select few), provide VMMC services at designated men’s clinics, allow men to select the gender of their service provider

At the community level and in schools, discussions with VMMC advisers and coaches were considered particularly helpful for VMMC clients or potential clients. One study surveyed South African men during VMMC services and found that 83% of men said they would not have been circumcised had they not received the intervention of motivational interviews with a VMMC adviser [34]. Similarly, one study with primarily adolescent clients showed that discussions with VMMC advisers and coaches were considered particularly helpful for VMMC clients or potential clients [31]. In schools, information and encouragement from peers was also valued [27]. At the facility and health system levels, mobile or other services that engaged men individually and reduced logistical barriers to VMMC were highly supported [35]. Including partners with clients in educational sessions provided opportunity for discussion on other issues, such as sexual and reproductive health [34]. Despite the wide acceptability of these interventions, one study noted concomitant HIV testing as a potential barrier to VMMC uptake [32].

### Values and preferences

No studies were identified for the review on end users’ and providers' values and preferences for the outcomes of interest.

### Costs

Eleven studies presented cost data [14,25,36–44], three of which were related to articles included in the main effectiveness review. Studies were published between 2012 and 2018 and took place in Kenya [38,41], South Africa [39,42,43], Tanzania [44], Uganda [36,37,40], and Zimbabwe [14,25]. Descriptions of included cost studies and their outcomes are presented in Table 5. Seven of the included studies assessed community-based interventions [14,36,40–44], two assessed school-based interventions [25,39], and two assessed facility-based interventions [37,38].

**Table 5:** Description of included studies–cost review.

Study	Location	Intervention	Cost findings
**Community-based**			
Alfonso et al., 2016 [36]	Uganda	Service modality: mobile vs fixed	Marginal cost per VMMC procedure: $23 (range $17–27) (mobile camp) vs $35 (range $35–40) (fixed service center) Cost in both service modalities primarily for wages and supplies Cost drivers: • increased number of surgeries led to increased cost of supplies • task shifting led to decreased wages • increased personnel experience led to increased efficiency
Awad et al., 2015 [14]	Zimbabwe	Age prioritization	# VMMCs needed to avert one infection: 10 (aged 20–24 years) vs 53 (aged 45–49 years) Cost per HIV infection averted: $811 (aged 20–24 years) vs $5,518 (aged 45–49 years)
Larson et al., 2015 [40]	Uganda	Service modality: mobile vs fixed	Cost of resources used per surgery: $61 (mobile, or $72 in more remote locations) vs $34 (fixed) Costs similar for: community mobilization, HIV testing, initial medical exam, VMMC staff Costs differed for: disposable surgical kits, additional upfront cost for mobile, number of staff Costs insensitive to patient flow over time
Marseille et al., 2014 [41]	Kenya	Service modality: “horizontal” (supporting mobile MOH teams to provide VMMC integrated with routine health services–assessed through the AIDS, Population, and Health Integrated Assistance Project (APHIA) II) vs “diagonal” (horizontal + vertical–dedicated project staff provide VMMC at additional outreach/mobile sites to supplement MOH services–assessed through the Nyanza Reproductive Health Society (NRHS))	Unit cost per adult VMMC: $38.62 ("horizontal") vs $44.24 ("diagonal") Unit cost per adult VMMC at base facilities: similar for both approaches Cost per HIV infection averted: $117.29 ("horizontal") vs $184.84 ("diagonal")
Soboil et al., 2012 [42]	South Africa	Service modality: mobile (roving, rural) vs fixed (specialized, urban)	Cost per VMMC procedure: $36 (mobile) vs $59 (fixed) Costs different in staffing (skeleton vs permanent team) and capital expenditure (no facilities vs fixed site) Support from Department of Health: mobile benefited from more support (interim staffing, provision of surgical consumables) Tracking client follow-up required greater time/resources in mobile than in fixed
Tchuenche et al., 2016 [43]	South Africa	Service modality: outreach vs fixed	Cost per VMMC procedure: $132 ($158 in public hospitals vs $121 in health centers/clinics) No substantial difference in cost at fixed sites vs fixed sites with outreach services Task shifting reduced labor costs by 17% (doctors to professional nurses)
Torres-Rueda et al., 2018 [44] (related to Wambura et al., 2017 [29] in effectiveness review)	Tanzania	Demand generation (age prioritization and outreach services)	Cost per VMMC procedure: $81.65 (intervention overall; $62 in Tabora and $130 in Njombe) vs $101.31 (control overall; $70 in Tabora and $191 in Njombe) Cost per HIV infection averted: $1424 (intervention in Tabora) / $2212 (intervention in Njombe) vs $1917 (control in Tabora) / $3018 (control in Njombe) Cost per DALY averted: $257 (intervention in Tabora) / $166 (intervention in Njombe) vs $354 (control in Tabora) / $224 (control in Njombe)
**School-based**			
George et al., 2017 [39] (related to Montague et al., 2014 [28] in effectiveness review)	South Africa	Age prioritization	Demand generation: Phase 2 (school outreach + transport to VMMC) vs Phase 3 (Phase 2 + peer recruiters + schedule change) Cost per VMMC procedure overall: $127.68 (32% demand creation, 10% HCT, 58% VMMC) Cost per VMMC procedure: $149.57 (Phase 2) (including demand creation $39.94 + VMMC $90.01) vs $110.85 (Phase 3) (including demand creation $41.65 + VMMC $60.60) More efficient utilization of clinic services in Phase 3
Kaufman et al., 2016 [25] (also in effectiveness review)	Zimbabwe	Age prioritization	Cost of "Make the Cut+" intervention: $1.99 per participant Costs included: training workshop and transportation for coaches, materials (t-shirt, printed curriculum, soccer balls, cones, laminated cards), coach stipend $3.50/half-day of work, 15% overhead Cost per additional VMMC client: $48.61
**Facility-based**			
Broughton et al., 2018 [37]	Uganda	Quality improvement (methods of disseminating information to facilities and training providers) - Manual alone: providing written manual - Manual and handover: manual plus handover meeting in which clinicians shared advice on implementing key changes and participated in group discussion - Manual, handover, and site visits: manual, handover meeting, and three site visits to facility where a coach provided individualized guidance and mentoring on improvement	Cost per patient: - $1.13 (Manual alone—small quality improvement gains) - $20.77 (Manual and handover—small quality improvement gains) - $28.83 (Manual, handover, and site visits—significant quality improvement gains)
Galarraga et al., 2017 [38]	Kenya	Economies of scale	Average cost per VMMC procedure: $66.30 (weighted mean by total annual patient volume: $41.10) - Doubling number of clients: 45% lower cost - Task shifting: 54% lower cost - Hospital vs non-hospital: no increased cost - Staff performance incentive: no increased cost - VMMC promoted through available male reproductive health services: 59% lower cost - Presence of community advisory board: 52% lower cost - Some activities performed outside facility: 49% higher cost - Facilities with highest performance: 67% higher cost - Facilities with highest performance AND highest competence: 90% lower cost (interaction)

DALY, disability-adjusted life year; MOH, Ministry of Health; VMMC, voluntary male medical circumcision

All monetary estimates are in US$.

Generally, cost studies showed that service delivery interventions may create economies of scale and efficiencies when implemented widely. Though activities to enhance uptake increase total costs, linkage to VMMC services that use task-shifting/sharing providers, pre-established facilities (both fixed site VMMC clinics and other clinics temporarily repurposed for VMMC services), and integrated HIV, sexual, and reproductive health services may save costs. Five studies assessing community-based interventions compared costs between various service delivery modalities, including mobile vs fixed, outreach vs fixed, and horizontal vs diagonal [36,40–43]. These studies had mixed results. One study comparing mobile and fixed service delivery in Uganda found that mobile services had a marginal cost per VMMC of US$23 (range: US$17 to US$27) compared with US$35 (range: US$35 to US$40) for fixed-site service delivery [36]. Another study in Uganda found that mobile services were in fact more expensive than fixed-site services (US$61–72 per mobile VMMC compared with US$34 per fixed VMMC) [40].

Overall, age prioritization interventions increased demand generation costs but resulted in sufficient demand to decrease overall VMMC unit costs and lower costs per HIV infection averted [14,25,39]. For example, one study assessing the cost of demand creation strategies targeting school-going adolescents in South Africa found that expanding in-school recruitment and adding peer recruiters while reducing VMMC clinic days increased overall demand creation unit costs but reduced VMMC procedure costs, thus reducing overall VMMC unit cost through increased recruitment and clinic efficiencies [39].

Finally, one study assessed cost effectiveness of three facility-based quality improvement strategies, which increased in intensity from providing a VMMC training manual to providing a training package including a manual, handover meeting, and individualized guidance and mentoring [37]. Quality was measured in terms of compliance to VMMC quality of care indices for four domains: informed consent, history-taking, anesthesia administration, and post-operative instructions. Cost of these strategies increased as intensity increased: the most intensive method cost US$28.83 per patient with large gains in improvement; the least intensive method was found to be most cost-efficient, at US$1.13 per patient with small quality improvement gains. A moderately intensive method cost $20.77 per patient but only yielded small quality improvement gains.

### Feasibility

Three studies with non-comparative designs were considered feasibility studies, all assessing facility-based interventions [45–47]. Dates of publication ranged from 2011 to 2014. Studies were from Kenya [45–47], South Africa [44], eSwatinti [46], Tanzania [45,46], and Zimbabwe [45]; two studies aggregated data from multiple countries [45,46]. These studies examined several intervention strategies, all community-based approaches to VMMC scale-up including campaigns and task-shifting/sharing. Descriptions of included feasibility studies and outcomes are presented in Table 6.

**Table 6:** Description of included studies–feasibility review.

Study	Location	Intervention	Feasibility findings
**Facility-based**			
Curran et al., 2011 [46]	Kenya, eSwatini, Tanzania	Scale-up Approaches to address human resources constraints	Improved VMMC uptake from: - efficiencies (surgical and non-surgical) - task shifting (VMMC counseling and HIV testing and counseling delegated to lay counselors; clinical officers and nurses replace doctors in all steps of VMMC surgery) - task sharing (some surgical steps delegated to non-physician clinicians, while physician maintains supervision of highest-level steps), especially when combined with approaches to improve layout and client flow of surgical services - temporary redeployment of public sector staff during VMMC campaign periods - expansion of the health workforce through recruitment of unemployed, recently retired, newly graduating, or on-leave health care workers - use of volunteer medical staff from other countries
Herman-Roloff et al., 2011 [47]	Kenya	Scale-up Task-shifting (medical or clinical officers to nurses) and HTS strategy	Task-shifting enabled more facilities to offer VMMC Proportion of VMMC clients who accepted HIV testing: <25% (client-initiated) vs >60% (provider-initiated)
Jennings et al., 2014 [45]	Kenya, South Africa, Tanzania, Zimbabwe	Scale-up	As the number of VMMC facilities increased, both facility preparedness (have guidelines on site, have equipment and supplies) and VMMC service quality (surgical tasks performed correctly, protective eyewear, safety tasks, post-operation monitoring and counseling) improved

HTS, HIV testing services; VMMC, voluntary male medical circumcision

Service delivery interventions were considered feasible, particularly regarding facility preparedness and health care workers. Facility preparedness and service quality improved as the number of VMMC facilities increased. Task-shifting and task-sharing enabled more facilities to offer VMMC (and also increased VMMC uptake and HIV testing), and scale-up increased both surgical and non-surgical efficiencies.

One study assessed facility preparedness and VMMC service quality in Kenya, South Africa, Tanzania, and Zimbabwe over the first year of VMMC scale-up [45]. It reported that facilities in Kenya and Zimbabwe dramatically improved service quality during scale-up, while facilities in South Africa and Tanzania maintained low scores and declined in some quality measures during scale-up.

Two studies highlighted human resource shortages as a potential barrier to the feasibility of VMMC scale-up in Kenya, Tanzania, and eSwatini; both studies reported that task-shifting and sharing were feasible strategies to mitigate these [46,47].

While these studies addressed general scale-up of VMMC, no studies were identified that assessed feasibility specifically of the interventions that sought to enhance uptake in settings where a higher level of VMMC scale-up had already occurred and more specific person-centered innovations were required.

## Discussion

A recent systematic review of barriers and facilitators to VMMC identified a number of personal, community, and service-level issues that could be addressed to improve VMMC uptake through service delivery interventions [48] but did not examine effectiveness. We identified nine studies from five countries in East and Southern Africa assessing how various changes to how health services were delivered could impact VMMC uptake. Our findings indicate that service delivery changes in the community, in schools, and at the facility level can improve VMMC uptake, including among adult men. We also found that effective interventions usually included several components. Specifically, interventions that used a combination of promotional, interpersonal and motivational communications along with changes to make service delivery more accessible generally had stronger effects than those using routine service delivery approaches only. For example, successful community-based interventions included home-based information and HIV testing services with interpersonal referral and follow-up to VMMC services and intensified VMMC demand creation efforts complementing periodic outreach and mobile services. Facility-based interventions included mentoring, enhanced sexual education, and improved provider competency. School-based interventions reduced access barriers to VMMC and included coach and peer mentoring support, particularly for adolescents; parents and community were also sensitized. Other intervention approaches may also yield improvements in VMMC uptake, but high-quality comparative data were not available to fully assess their impact in this review.

While service delivery interventions generally increased VMMC uptake, overall uptake following some interventions remained low. This may reflect the time it takes for those exposed to interventions to decide to pursue VMMC and undergo the procedure. For example, a qualitative study of behavior change pathways to VMMC in Zimbabwe highlighted the dynamic nature of demand for VMMC, which involves a man’s progression through multiple stages of change over time [49]. Indeed, in our review, studies with longer follow-up periods typically reported higher uptake overall. Differences in follow-up time across the included studies (with follow-up periods ranging from one to 38 months for uptake of VMMC) posed additional challenges for comparison.

Although we grouped interventions into three categories (community- (including home-), school-, and facility-based) for this review, we found a wide range of intervention approaches within these categories. The diversity of interventions and limited replication of intervention strategies in multiple settings made it difficult to compare interventions or to assess the true effect and feasibility of interventions that appear to be promising. Four similar studies were undertaken in Tanzania; all found that providing periodic outreach campaigns increased uptake. Though VMMC programmes and implementing partners in priority countries have employed diverse service delivery interventions to achieve targets and improve VMMC coverage, we were only able to find published studies evaluating such interventions in five countries: South Africa, Tanzania, Uganda, Zambia, and Zimbabwe. This may partially reflect the challenges of creating locally relevant, people-centered service delivery approaches or the infrastructure needed to undertake research and publish peer-reviewed intervention evaluations.

The majority of interventions identified in the effectiveness review were community-based and included changes to VMMC referral approaches with enhanced follow up, intensified demand creation efforts with community-based services closer to work or home, campaigns, mobile services, and use of technology to strategically plan service delivery for hard-to-reach locations. Campaigns were more effective in increasing VMMC uptake among adolescents than adult men, who responded more to interpersonal communication-based approaches. Because VMMC uptake is generally higher among adolescents, it could be that older men feel less comfortable accessing VMMC in high-volume settings or have unique concerns that are better addressed by tailored communication. For example, a qualitative study of social and individual factors influencing VMMC decisions among adult men in Tanzania found that men often expressed shame at seeking services with younger boys [50]. This study also found that men had concerns related to the post-surgical period that may not be shared by adolescents, including concerns about required abstinence and lost income. The focus on community-based service delivery interventions over school- or facility-based interventions is similarly unsurprising, given the focus on reaching adult men with VMMC in recent years [7,9,51] and the disproportionately high uptake of VMMC among younger clients, especially adolescent boys who are likely to be in school [6,8,18,51–53].

However, we only identified five studies that focused on reaching specific age groups with VMMC. Two of these, both in Tanzania, aimed to reach adult clientele (age >20) with VMMC; these studies had somewhat mixed results but showed that periodic mobile service delivery and interpersonal motivational advisors/mentors were promising strategies for enhancing uptake among adult men. The remaining three studies with age-targeting focused on school-based interventions, which unsurprisingly yielded greater uptake among adolescents (10–19 years) compared with adult men; these approaches relied on trusted mentors and facilitated access to clinics. In addition, two studies of VMMC services in the general male population (i.e., no age-targeted services) provided age-disaggregated uptake data; consistent with the literature [6–9], both demonstrated increased uptake among younger compared with adult men. The other three studies in this review did not address age-targeting strategies.

While we found data for the effectiveness review on VMMC uptake, we found limited data on uptake of HIV testing within VMMC services, and no comparative data on uptake of safer sex and risk reduction counseling within VMMC services. HIV testing and safer sex and risk reduction counseling are considered standard parts of HIV testing services, so researchers may not have measured these components of VMMC separately. While these measures are a potential measure of service quality, this is also challenging, as individual clients may opt-out of these services, and 100% uptake on either of these measures might indicate coercive or non-optional uptake. Further, despite the lack of studies quantifying uptake of counseling during VMMC, relationships between VMMC, counseling, and risk compensation have been assessed substantially in the literature; evidence indicates that VMMC leads to little or no risk compensation in sub-Saharan Africa [54–60].

Reviews of VMMC acceptability, cost, and feasibility identified studies from seven countries and found promising results. Findings from the acceptability review suggest that service delivery approaches were generally considered acceptable and were perceived to help men choose to get circumcised. However, service delivery interventions that do not adequately consult or partner with community members were met with less acceptance.

Findings from the cost review indicate that mobile and outreach services may reduce VMMC unit cost, compared with fixed services. Further, while age prioritization interventions were shown to increase demand generation costs, they also resulted in sufficient demand to decrease overall VMMC unit costs and lower costs per HIV infection averted. While we found few studies assessing the feasibility of VMMC service delivery interventions, findings from our feasibility review suggest that task-shifting or sharing and facility preparedness play an important role in intervention success. These are important considerations for future service delivery interventions.

Strengths of this review include our attention to a range of factors relevant to policy and programmatic decision-making beyond intervention effectiveness, including values and preferences, acceptability, cost, and feasibility. We also used rigorous search, screening, data extraction, and analysis methods to identify all possible articles and conference abstracts that met our inclusion criteria. However, our search did not include grey literature or program reports, which may have provided valuable additional information. Many VMMC programs may be implementing service delivery strategies without rigorous or published evaluations. We organized our findings for the effectiveness review by study design to distinguish between the quality of evidence of RCTs compared with observational studies, but the diversity of included articles, populations, study designs, and outcomes made it difficult to synthesize results. Further, because several included articles used a combination of intervention approaches—and some of these included a control or comparison group also receiving some element of the intervention—we were limited in our ability to precisely conclude which intervention approaches were responsible for reported effects. Additional research should consider further assessing the differential impacts as well as the impact of age- or HIV risk-prioritization interventions on uptake among subgroups.

## Conclusions

Findings from this systematic review indicate that changes to service delivery to increase VMMC uptake among a target population, specifically adult men or adolescent boys, can be effective, cost-efficient, and acceptable. These various intervention strategies, including interpersonal mentorship/advisor support, targeted demand-generation activities to increase awareness and intent, with improved service access by periodic VMMC campaigns, mobile VMMC service delivery, facilitated transportation, and community (and parent for adolescents) engagement seem to increase uptake of VMMC. But their full impact is unclear due to a lack of published, high-quality evaluations of such interventions. As VMMC programs continue striving to meet targets and better engage men, there is increased need to identify cost-effective and efficient strategies that are accessible and acceptable to those at highest risk and harder to reach. While further evaluations are needed, given the time to undertake such evaluations, approaches using program data should be improved to guide changes in interventions, partnering with men and communities. Given the challenges other HIV services have had reaching men in sub-Saharan Africa, the diverse and creative approaches to offering services presented in this review represent promising opportunities for VMMC and other HIV service provision.

## Supporting information

S1 Table Quality assessment of included studies–effectiveness review. (DOCX)

S1 File PRISMA checklist. (DOC)

## References

[1] Auvert B, Taljaard D, Lagarde E, Sobngwi-Tambekou J, Sitta R, Puren A. Randomized, controlled intervention trial of male circumcision for reduction of HIV infection risk: the ANRS 1265 Trial. PLoS Med. 2005;2(2):1549–676. 10.1371/journal.pmed.0020298 PMC1262556 16231970

[2] Bailey RC, Moses S, Parker CB, Agot K, Maclean I, Krieger JN, et al Male circumcision for HIV prevention in young men in Kisumu, Kenya: a randomised controlled trial. Lancet. 2007;369(9562):643–56. 10.1016/S0140-6736(07)60312-2 17321310

[3] Gray RH, Kigozi G, Serwadda D, Makumbi F, Watya S, Nalugoda F, et al Male circumcision for HIV prevention in men in Rakai, Uganda: a randomised trial. Lancet. 2007;369(9562):657–66. 10.1016/S0140-6736(07)60313-4 17321311

[4] WHO, UNAIDS. WHO/UNAIDS technical consultation on male circumcision and HIV prevention: research implications for policy and programming–conclusions and recommendations (6–8 March 2007). Montreux, Switzerland: World Health Organization and Joint United Nations Programme on HIV/AIDS, 2007.

[5] WHO. Voluntary medical male circumcision for HIV prevention: Progress brief. Geneva, Switzerland: World Health Organization, 2018.

[6] Patel EU, Kaufman MR, Dam KH, Van Lith LM, Hatzold K, Marcell AV, et al Age differences in perceptions of and motivations for voluntary medical male circumcision among adolescents in South Africa, Tanzania, and Zimbabwe. Clin Infect Dis. 2018;66(Suppl 3):S173–S82. 10.1093/cid/cix951 29617775 PMC5888947

[7] Kripke K, Njeuhmeli E, Samuelson J, Schnure M, Dalal S, Farley T, et al Assessing progress, impact, and next steps in rolling out voluntary medical male circumcision for HIV prevention in 14 priority countries in Eastern and Southern Africa through 2014. PLoS One. 2016;11(7):e0158767 10.1371/journal.pone.0158767 27441648 PMC4955652

[8] Njeuhmeli E, Hatzold K, Gold E, Mahler H, Kripke K, Seifert-Ahanda K, et al Lessons learned from scale-up of voluntary medical male circumcision focusing on adolescents: benefits, challenges, and potential opportunities for linkages with adolescent HIV, sexual, and reproductive health services. J Acquir Immune Defic Syndr. 2014;66(Suppl 2):S193–S9. 24918595 10.1097/QAI.0000000000000179

[9] WHO, UNAIDS. Joint strategic action framework to accelerate the scale-up of voluntary medical male circumcision for HIV prevention in Eastern and Southern Africa, 2012–2016. Geneva, Switzerland: World Health Organization and Joint United Nations Programme on HIV/AIDS, 2011.

[10] Toledo C, Davis SM, Thomas A, Kiggundu V, Cooney C, Watts DH. Making a pivot to maximize impact: age of clients at PEPFAR-supported voluntary medical male circumcision programs for HIV prevention, 2017. International AIDS Conference; Amsterdam, Netherlands: July 2018.

[11] Kennedy CE, Yeh PT, Atkins K, Fonner VA, Sweat MD, O'Reilly K, et al Economic incentives to increase uptake of voluntary medical male circumcision for HIV prevention: A systematic review and meta-analysis. PLoS One. [Manuscript under review]. 10.1371/journal.pone.0227623 PMC6961886 31940422

[12] Moher D, Liberati A, Tetzlaff J, Altman DG. Preferred reporting items for systematic reviews and meta-analyses: the PRISMA statement. Ann Intern Med. 2009;151(4):264–9. 10.7326/0003-4819-151-4-200908180-00135 19622511

[13] WHO. Models to inform fast tracking voluntary medical male circumcision in HIV combination prevention. Geneva, Switzerland: World Health Organization, 2017.

[14] Awad SF, Sgaier SK, Ncube G, Xaba S, Mugurungi OM, Mhangara MM, et al A Reevaluation of the voluntary medical male circumcision scale-up plan in Zimbabwe. PLoS One. 2015;10(11):e0140818 10.1371/journal.pone.0140818 26529596 PMC4646702

[15] Awad SF, Sgaier SK, Tambatamba BC, Mohamoud YA, Lau FK, Reed JB, et al Investigating voluntary medical male circumcision program efficiency gains through subpopulation prioritization: insights from application to Zambia. PLoS One. 2015;10(12):e0145729 10.1371/journal.pone.0145729 26716442 PMC4696770

[16] Kripke K, Opuni M, Schnure M, Sgaier S, Castor D, Reed J, et al Age Targeting of Voluntary Medical Male Circumcision Programs Using the Decision Makers’ Program Planning Toolkit (DMPPT) 2.0. PLoS One. 2016;11(7):e0156909 10.1371/journal.pone.0156909 27410966 PMC4943717

[17] O'Neill J, Tabish H, Welch V, Petticrew M, Pottie K, Clarke M, et al Applying an equity lens to interventions: using PROGRESS ensures consideration of socially stratifying factors to illuminate inequities in health. J Clin Epidemiol. 2014;67(1):56–64. 10.1016/j.jclinepi.2013.08.005 24189091

[18] Carrasco MA, Grund JM, Davis SM, Ridzon R, Mattingly M, Wilkinson J, et al Systematic review of the effect of economic compensation and incentives on uptake of voluntary medical male circumcision among men in sub-Saharan Africa. AIDS Care. 2018;30(9):1071–82. 10.1080/09540121.2018.1453921 29566546 PMC6800131

[19] Higgins JPT, Green S. Chapter 8.5 The Cochrane Collaboration's tool for assessing risk of bias In: Higgins JPT, Altman DG, Sterne JAC, on behalf of the Cochrane Statistical Methods Group and the Cochrane Bias Methods Group, editors. Cochrane Handbook for Systematic Reviews of Interventions, Version 5-1-0 [updated March 2011]. London, England: The Cochrane Collaboration; 2011.

[20] Kennedy CE, Fonner VA, Armstrong KA, Denison JA, Yeh PT, O'Reilly KR, et al The Evidence Project risk of bias tool: assessing study rigor for both randomized and non-randomized intervention studies. Syst Rev. 2019;8(1):3 10.1186/s13643-018-0925-0 30606262 PMC6317181

[21] WHO. WHO Handbook of Guideline Development. Geneva, Switzerland: World Health Organization; 2012.

[22] Ashengo TA, Hatzold K, Mahler H, Rock A, Kanagat N, Magalona S, et al Voluntary medical male circumcision (VMMC) in Tanzania and Zimbabwe: service delivery intensity and modality and their influence on the age of clients. PLoS One. 2014;9(5):e83642 10.1371/journal.pone.0083642 24801882 PMC4011872

[23] Barnabas RV, van Rooyen H, Tumwesigye E, Brantley J, Baeten JM, van Heerden A, et al Uptake of antiretroviral therapy and male circumcision after community-based HIV testing and strategies for linkage to care versus standard clinic referral: a multisite, open-label, randomised controlled trial in South Africa and Uganda. Lancet HIV. 2016;3(5):e212–20. 10.1016/S2352-3018(16)00020-5 27126488 PMC4852382

[24] Hellar AM, Boyee D, Mahler H, Plotkin M, Ng'wanakilala T, Curran K, et al Mobile VMMC teams in Tanzania see older clients and have higher followup rates. Top Antivir Med. 2015;23(e-1):502.

[25] Kaufman ZA, DeCelles J, Bhauti K, Hershow RB, Weiss HA, Chaibva C, et al A sport-based intervention to increase uptake of voluntary medical male circumcision among adolescent male students: results From the MCUTS 2 cluster-randomized trial in Bulawayo, Zimbabwe. J Acquir Immune Defic Syndr. 2016;72(Suppl 4):S292–8. 27404011 10.1097/QAI.0000000000001046 PMC5054964

[26] Mahler H, Searle S, Plotkin M, Kulindwa Y, Greenberg S, Mlanga E, et al Covering the last kilometer: using GIS to scale-up voluntary medical male circumcision services in Iringa and Njombe regions, Tanzania. Glob Health Sci Pract. 2015;3(3):503–15. 10.9745/GHSP-D-15-00151 26374807 PMC4570020

[27] Miiro G, DeCelles J, Rutakumwa R, Nakiyingi-Miiro J, Muzira P, Ssembajjwe W, et al Soccer-based promotion of voluntary medical male circumcision: A mixed-methods feasibility study with secondary students in Uganda. PLoS One. 2017;12(10):e0185929 10.1371/journal.pone.0185929 29016651 PMC5633183

[28] Montague C, Ngcobo N, Mahlase G, Frohlich J, Pillay C, Yende-Zuma N, et al Implementation of adolescent-friendly voluntary medical male circumcision using a school based recruitment program in rural KwaZulu-Natal, South Africa. PLoS One. 2014;9(5):e96468 10.1371/journal.pone.0096468 24788339 PMC4008624

[29] Wambura M, Mahler H, Grund J, Larke N, Mshana G, Kuringe E, et al Increasing voluntary medical male circumcision uptake among adult men in Tanzania. Aids. 2017;31(7):1025–34. 10.1097/QAD.0000000000001440 28350578 PMC5378002

[30] Weiss SM, Zulu R, Jones DL, Redding CA, Cook R, Chitalu N. The Spear and Shield intervention to increase the availability and acceptability of voluntary medical male circumcision in Zambia: a cluster randomised controlled trial. Lancet HIV. 2015;2(5):e181–9. 10.1016/S2352-3018(15)00042-9 26120594 PMC4478609

[31] DeCelles J, Hershow RB, Kaufman ZA, Gannett KR, Kombandeya T, Chaibva C, et al Process evaluation of a sport-based voluntary medical male circumcision demand-creation intervention in Bulawayo, Zimbabwe. J Acquir Immune Defic Syndr. 2016;72(Suppl 4):S304–S8. 27749598 10.1097/QAI.0000000000001172 PMC5054959

[32] Katisi M, Daniel M. Safe male circumcision in Botswana: tension between traditional practices and biomedical marketing. Glob Public Health. 2015;10(5–6):739–56. 10.1080/17441692.2015.1028424 25866013 PMC4487566

[33] Kilima SP, Shayo EH, Msovela J, Senkoro KP, Mayala BK, Mboera LEG, et al The potential of involving traditional practitioners in the scaling up of male circumcision in the context of HIV prevention in Tanzania. Tanzania J Health Res. 2012;14(1):48–60. 10.4314/thrb.v14i1.9 26591747

[34] Marshall E, Rain-Taljaard R, Tsepe M, Monkwe C, Taljaard D, Hlatswayo F, et al Obtaining a male circumcision prevalence rate of 80% among adults in a short time: An observational prospective intervention study in the Orange Farm township of South Africa. Med. 2017;96(4):e5328. 10.1097/MD.0000000000005328 PMC5287938 28121914

[35] Semo BW, Wirth KE, Ntsuape C, Barnhart S, Kleinman NJ, Ramabu N, et al Modifying the health system to maximize voluntary medical male circumcision uptake: a qualitative study in Botswana. HIV AIDS (Auckl). 2018;10:1–8. 29296100 10.2147/HIV.S144407 PMC5739115

[36] Alfonso YN, Bishai D, Nantongo A, Kakembo R, Kobusinge S, Kacker S, et al Trends in the marginal cost of male circumcision in rural Rakai Uganda. J Acquir Immune Defic Syndr. 2016;73(5):564–71. 10.1097/QAI.0000000000001144 27509246 PMC5110383

[37] Broughton EI, Karamagi E, Kigonya A, Lawino A, Marquez L, Lunsford SS, et al The cost-effectiveness of three methods of disseminating information to improve medical male circumcision in Uganda. PLoS One. 2018;13(4):e0195691 10.1371/journal.pone.0195691 29672578 PMC5908073

[38] Galárraga O, Wamai RG, Sosa-Rubí SG, Mugo MG, Contreras-Loya D, Bautista-Arredondo S, et al HIV prevention costs and their predictors: evidence from the ORPHEA Project in Kenya. Health Policy Plan. 2017;32(10):1407–16. 10.1093/heapol/czx121 29029086 PMC5886164

[39] George G, Strauss M, Asfaw E. The cost of demand creation activities and voluntary medical male circumcision targeting school-going adolescents in KwaZulu-Natal, South Africa. PLoS One. 2017;12(6):e0179854 10.1371/journal.pone.0179854 28632768 PMC5478150

[40] Larson B, Tindikahwa A, Mwidu G, Kibuuka H, Magala F. How much does it cost to improve access to voluntary medical male circumcision among high-risk, low-income communities in Uganda? PLoS One. 2015;10(3):e0119484 10.1371/journal.pone.0119484 25774677 PMC4361173

[41] Marseille E, Kahn JG, Beatty S, Jared M, Perchal P. Adult male circumcision in Nyanza, Kenya at scale: the cost and efficiency of alternative service delivery modes. BMC Health Serv Res. 2014;14:31 10.1186/1472-6963-14-31 24450374 PMC3902184

[42] Soboil N, Cockburn J, Rech D, Taljaard D. A comparative analysis of two high-volume male medical circumcision (MMC) operational models with similar service delivery outcomes in different settings within Gauteng and KwaZulu-Natal provinces in South Africa: Urban Centre for HIV/AIDS Prevention Studies (CHAPS) versus rural-SACTWU Worker Health Program (SWHP). J Int AIDS Soc. 2012;15(S3):239.

[43] Tchuenche M, Palmer E, Haté V, Thambinayagam A, Loykissoonlal D, Njeuhmeli E, et al The cost of voluntary medical male circumcision in South Africa. PLoS One. 2016;11(10):e0160207 10.1371/journal.pone.0160207 27783612 PMC5082632

[44] Torres-Rueda S, Wambura M, Weiss HA, Plotkin M, Kripke K, Chilongani J, et al Cost and cost-effectiveness of a demand creation intervention to increase uptake of voluntary medical male circumcision in Tanzania: spending more to spend less. J Acquir Immune Defic Syndr. 2018;78(3):291–9. 10.1097/QAI.0000000000001682 29557854 PMC6012046

[45] Jennings L, Bertrand J, Rech D, Harvey SA, Hatzold K, Samkange CA, et al Quality of voluntary medical male circumcision services during scale-up: a comparative process evaluation in Kenya, South Africa, Tanzania and Zimbabwe. PLoS One. 2014;9(5):e79524 10.1371/journal.pone.0079524 24801073 PMC4011679

[46] Curran K, Njeuhmeli E, Mirelman A, Dickson K, Adamu T, Cherutich P, et al Voluntary medical male circumcision: strategies for meeting the human resource needs of scale-up in southern and eastern Africa. PLoS Med. 2011;8(11):e1001129 10.1371/journal.pmed.1001129 22140364 PMC3226463

[47] Herman-Roloff A, Llewellyn E, Obiero W, Agot K, Ndinya-Achola J, Muraguri N, et al Implementing voluntary medical male circumcision for HIV prevention in Nyanza Province, Kenya: lessons learned during the first year. PLoS One. 2011;6(4):e18299 10.1371/journal.pone.0018299 21483697 PMC3070734

[48] Carrasco MA, Wilkinson J, Kasdan B, Fleming P. Systematic review of barriers and facilitators to voluntary medical male circumcision in priority countries and programmatic implications for service uptake. Glob Public Health. 2019;14(1):91–111. 10.1080/17441692.2018.1465108 29695201

[49] Price JE, Phiri L, Mulenga D, Hewett PC, Topp SM, Shiliya N, et al Behavior change pathways to voluntary medical male circumcision: narrative interviews with circumcision clients in Zambia. PLoS One. 2014;9(11):e111602 10.1371/journal.pone.0111602 25375790 PMC4222873

[50] Plotkin M, Castor D, Mziray H, Küver J, Mpuya E, Luvanda PJ, et al “Man, what took you so long?” Social and individual factors affecting adult attendance at voluntary medical male circumcision services in Tanzania. 2013;1(1):108–16. 10.9745/GHSP-D-12-00037 PMC4168557 25276521

[51] Health Communication Capacity Collaborative (HC3). Technical considerations for demand generation for voluntary medical male circumcision in the context of the age pivot. Baltimore, Maryland: Johns Hopkins Center for Communication Programs (CCP), 2016.

[52] Hatzold K, Mavhu W, Jasi P, Chatora K, Cowan FM, Taruberekera N, et al Barriers and motivators to voluntary medical male circumcision uptake among different age groups of men in Zimbabwe: results from a mixed methods study. PLoS One. 2014;9(5):e85051 10.1371/journal.pone.0085051 24802746 PMC4011705

[53] Haacker M, Fraser-Hurt N, Gorgens M. Effectiveness of and financial returns to voluntary medical male circumcision for HIV prevention in South Africa: an incremental cost-effectiveness analysis. PLoS Med. 2016;13(5):e1002012 10.1371/journal.pmed.1002012 27138961 PMC4854479

[54] Shi C-F, Li M, Dushoff J. Evidence that promotion of male circumcision did not lead to sexual risk compensation in prioritized Sub-Saharan countries. PLoS One. 2017;12(4):e0175928 10.1371/journal.pone.0175928 28441458 PMC5404849

[55] Govender K, George G, Beckett S, Montague C, Frohlich J. Risk Compensation Following Medical Male Circumcision: Results from a 1-Year Prospective Cohort Study of Young School-Going Men in KwaZulu-Natal, South Africa. Int J Behav Med. 2018;25(1):123–30. 10.1007/s12529-017-9673-0 28688094

[56] Westercamp N, Agot K, Jaoko W, Bailey RC. Risk compensation following male circumcision: results from a two-year prospective cohort study of recently circumcised and uncircumcised men in Nyanza Province, Kenya. AIDS Behav. 2014;18(9):1764–75. 10.1007/s10461-014-0846-4 25047688

[57] Westercamp M, Jaoko W, Mehta S, Abuor P, Siambe P, Robert CB. Changes in male circumcision prevalence and risk compensation in the Kisumu, Kenya population 2008–2013. Journal of Acquired Immune Deficiency Syndromes and Human Retrovirology. 2017;74(2):e30. 10.1097/QAI.0000000000001180 PMC5233580 27632232

[58] L’Engle K, Lanham M, Loolpapit M, Oguma I. Understanding partial protection and HIV risk and behavior following voluntary medical male circumcision rollout in Kenya. Health Educ Res. 2013;29(1):122–30. 10.1093/her/cyt103 24293524 PMC3894669

[59] Mukudu H, Dietrich J, Otwombe K, Manentsa M, Hlongwane K, Haas-Kogan M, et al Voluntary medical male circumcision (VMMC) for prevention of heterosexual transmission of HIV and risk compensation in adult males in Soweto: Findings from a programmatic setting. PLoS One. 2019;14(3):e0213571 10.1371/journal.pone.0213571 30845185 PMC6405100

[60] Ortblad KF, Harling G, Chimbindi N, Tanser F, Salomon JA, Bärnighausen T. Does incident circumcision lead to risk compensation? Evidence from a population cohort in KwaZulu-Natal, South Africa. JAIDS. 2019;80(3):269–75. 10.1097/QAI.0000000000001912 30531298 PMC6375765

