# Supplementary material for: Service delivery interventions to increase uptake of voluntary medical male circumcision for HIV prevention: A systematic review
Source: PLoS One. 2020 Jan 13;15(1):e0227755. doi: 10.1371/journal.pone.0227755 (PMC6957297; doi:10.1371/journal.pone.0227755)
Supplement: S1 Table — (DOCX) [file pone.0227755.s001.docx]

**Service delivery interventions to increase uptake of voluntary medical male circumcision for HIV prevention: A systematic review**

**S1 Table.** Quality assessment of included studies – effectiveness review.

| Cochrane risk of bias tool for randomized controlled trials.[19] | | | | | | | | | | |
| --- | --- | --- | --- | --- | --- | --- | --- | --- | --- | --- |
| Study | Type of bias | | | Judgment | Support for Judgment | | | | | |
| Barnabas et al., 2016 | Random sequence generation (selection bias) | | | Low risk | “HIV-negative uncircumcised men were randomly assigned to receive mobile phone text message reminders, home visits, or standard referral. The study biostatistician (JPH) generated the randomization schedule by use of a computer-generated random number program with varying block sizes ( … multiples of three for participants in the circumcision linkage arms) stratified by country.” | | | | | |
|  | Allocation concealment (selection bias) | | | Low risk | “The randomization allocation was automatically assigned by the computer software to the study participant after informed consent was obtained. Once eligibility was assessed and the participant consented to continuing with the study, the randomisation assignment was revealed. Study staff did not have access to the randomisation code.” | | | | | |
|  | Blinding of participants and personnel (performance bias) | | | Low risk | Blinding of participants and personnel not possible due to study design, but outcomes (VMMC uptake at 3 and 9 months, social harms) not likely to be influenced by lack of blinding. | | | | | |
|  | Blinding of outcome assessment (detection bias) | | | Low risk | Blinding of outcomes not possible due to study design, but outcomes (VMMC uptake at 3 and 9 months, social harms) not likely to be influenced by lack of blinding. | | | | | |
|  | Incomplete outcome data addressed (attrition bias) | | | Low risk | Reasons for missing outcome data unlikely to be related to true outcome; censoring unlikely to be introducing bias | | | | | |
|  | Selective reporting (reporting bias) | | | Unclear risk | Data on 3-month VMMC uptake (primary outcome) and social harms (secondary outcome) are reported but data on 9-month uptake (secondary outcome) are not: “This effect of increased uptake of male circumcision with text messaging promotion and lay counsellor visits was similar and sustained at the month 9 exit visit, and did not change when we excluded participants before confirmation of text message delivery.” | | | | | |
|  | Other bias | | | High risk | “After 448 participants had been enrolled, we discovered that the mobile phone companies were blocking the text messages and participants were not receiving the intervention. The study biostatistician redid the block randomisation code to increase the number of participants randomly assigned to the text message group to ensure at least 80% power for a 10% effect of the male circumcision linkage strategies.” | | | | | |
| Kaufman et al., 2016 | Random sequence generation (selection bias) | | | Low risk | “Schools were the unit of randomization … Within each stratum [private schools, large public schools (>=300 students enrolled), and small public schools (<300 students)], schools were randomized in a 1:1 ratio at a public event held in Bulawayo. For each stratum, each school’s name was written on a piece of paper. Papers were then blindly drawn from a concealed bucket to assign them to the intervention or control group.” | | | | | |
|  | Allocation concealment (selection bias) | | | Unclear risk | Method of concealment is not described or not described in sufficient detail to allow a definite judgement: “Papers were then blindly drawn from a concealed bucket.” | | | | | |
|  | Blinding of participants and personnel (performance bias) | | | Low risk | Blinding of participants and personnel not possible due to study design, but primary outcome (VMMC uptake from 07/03/2014 to 06/07/2014) not likely to be influenced by lack of blinding. | | | | | |
|  | Blinding of outcome assessment (detection bias) | | | Low risk | Blinding of outcomes not possible due to study design, but primary outcome (VMMC uptake from 07/03/2014 to 06/07/2014) not likely to be influenced by lack of blinding. | | | | | |
|  | Incomplete outcome data addressed (attrition bias) | | | Low risk | Reasons for missing outcome data unlikely to be related to true outcome; censoring unlikely to be introducing bias | | | | | |
|  | Selective reporting (reporting bias) | | | Low risk | The study protocol is not available but it is clear that the published reports include all expected outcomes, including the primary outcome that was pre-specified: “VMMC uptake from 07/03/2014 to 06/07/2014.” | | | | | |
|  | Other bias | | | Low risk | The study appears to be free of other sources of bias. | | | | | |
| Wambura et al., 2017 | Random sequence generation (selection bias) | | | Low risk | “The allocation sequence was computer-generated with a 1:1 allocation ratio within region.” | | | | | |
|  | Allocation concealment (selection bias) | | | Low risk | “The allocation was unmasked to the field teams delivering the intervention and control strategies and to field staff collecting outcome data. Study participants were masked to study arm.” | | | | | |
|  | Blinding of participants and personnel (performance bias) | | | Low risk | Blinding of participants and personnel not possible due to study design, but primary outcome (proportion of VMMC clients aged 20-34) not likely to be influenced by lack of blinding. | | | | | |
|  | Blinding of outcome assessment (detection bias) | | | Low risk | Blinding of outcomes not possible due to study design, but primary outcome (proportion of VMMC clients aged 20-34) not likely to be influenced by lack of blinding. | | | | | |
|  | Incomplete outcome data addressed (attrition bias) | | | Low risk | No participant loss; no missing outcome data. | | | | | |
|  | Selective reporting (reporting bias) | | | Low risk | The study protocol is not available but it is clear that the published reports include all expected outcomes, including those that were pre-specified. (“The primary outcome was the proportion of VMMC clients aged 20–34 years. Secondary outcomes were the proportion of clients aged at least 20 years, mean number of clients per cluster aged 20–34 years, and mean number of clients per cluster of all ages.”) | | | | | |
|  | Other bias | | | Low risk | The study appears to be free of other sources of bias. | | | | | |
| Weiss et al., 2015 | Random sequence generation (selection bias) | | | Low risk | “Randomisation was done by Zambian investigators with a random allocation computer-generated sequence; trial statisticians did not participate in randomisation.” | | | | | |
|  | Allocation concealment (selection bias) | | | Low risk | “Clinics were notified of their condition assignment with sealed envelopes.” | | | | | |
|  | Blinding of participants and personnel (performance bias) | | | Low risk | Blinding of participants and personnel not possible due to study design, but outcomes (uptake of VMMC by 12 months post-intervention and condom use after VMMC) not likely to be influenced by lack of blinding. Outcomes measured using health facility data. | | | | | |
|  | Blinding of outcome assessment (detection bias) | | | Low risk | Blinding of outcomes not possible due to study design, but outcomes (uptake of VMMC by 12 months post-intervention and condom use after VMMC) not likely to be influenced by lack of blinding. | | | | | |
|  | Incomplete outcome data addressed (attrition bias) | | | Low risk | Reasons for missing outcome data unlikely to be related to true outcome; censoring unlikely to be introducing bias | | | | | |
|  | Selective reporting (reporting bias) | | | Low risk | The study protocol is not available but it is clear that the published reports include all expected outcomes, including the primary outcomes that were pre-specified: “likelihood of voluntary medical male circumcision by 12 months post-intervention, and condom use after voluntary medical male circumcision among participants receiving the intervention.” | | | | | |
|  | Other bias | | | Low risk | The study appears to be free of other sources of bias. | | | | | |
| Evidence Project risk of bias tool for non-randomized studies.[20] | | | | | | | | | | |
|  | **Study design includes** | | | **Participant representativeness** | | | | | **Comparison groups equivalent at baseline on** | |
|  | **Cohort** | **Control or comparison group** | **Pre/post intervention data** | **Participants randomly assigned to intervention** | | **Participants randomly selected for assessment** | **Control for potential confounders** | **Follow-up rate ≥ 75%** | **Socio-demographics** | **Outcome measures** |
| Ashengo et al., 2014 | No | Yes | No | No | | No | No | NA | NR | NR |
| Hellar et al., 2015 | No | Yes | No | No | | No | No | NA | NR | NR |
| Mahler et al., 2015 | No | No | Yes | No | | No | No | NA | NA | NA |
| Miiro et al., 2017 | No | Yes | Yes | No | | No | No | NA | NR | NR |
| Montague et al., 2014 | No | Yes | Yes | No | | No | No | NA | NR | NR |
